# Assessment of sodium thiosulfate neutralizing effect on micro-hardness of dentin treated with sodium hypochlorite

**DOI:** 10.1186/s12903-020-01320-2

**Published:** 2020-11-12

**Authors:** Safoora Sahebi, Fereshteh Sobhnamayan, Fariborz Moazami, Mohammadhasan Naseri

**Affiliations:** Department of Endodontics, Dental School, Shiraz University of Medical Sciences, Ghasrdasht Street, 71956-15878 Shiraz, Iran

**Keywords:** Dentin, Micro-hardness tests, Sodium hypochlorite, Sodium thiosulfate

## Abstract

**Background:**

This study aims to evaluate the ability of sodium thiosulfate (STS) to neutralize the adverse effect of sodium hypochlorite (NaOCl) on dentin micro-hardness.

**Methods:**

Fifty single-rooted teeth were longitudinally sectioned. The samples divided into a control and four sample groups (n = 20). All the samples were immersed in different solutions as follows, Control: Normal saline for 15 min, G1and G2: 2.5% NaOCl for 15 min, G3: 2.5% NaOCl for 15 min, followed by 5% STS for 10 min, G4: Normal saline for 15 min followed by 5% STS for 10 min. All groups except G1 incubated for one week before the test. The micro-hardness of samples was measured. Data were analyzed using the Kruskal–Wallis test for pairwise comparisons. A *p* value < 0.05 was considered significant**.**

**Results:**

All groups showed a significant decrease in the micro-hardness value compared with the control group. NaOCl for one week (G2) reduced the micro-hardness of dentine compared with samples, tested immediately after immersion in NaOCl (G1) (*p* < 0.05). NaOCl alone (G2) or treated with STS (G3) resulted in a significant decrease in micro-hardness compared with the STS group (G4) (*p* < 0.05).

**Conclusions:**

STS as a neutralizing agent could not prevent the dentin micro-hardness downturn caused by NaOCl.

## Background

Irrigants and intra-canal medicaments such as NaOCl and calcium hydroxide may have some adverse effects on the physical and mechanical characteristics of dentin, result in reducing flexural strength, micro-hardness, and modulus of elasticity [1–5]. NaOCl is undeniably the most widely used irrigant in endodontics because of its efficacy against microorganisms and its ability to dissolve organic tissues [6, 7]. Besides its benefits to other irrigation solutions, NaOCl could dissolve some of the organic parts of dentinal tissue as well [8]. This proteolytic effect caused a 30% weakening of root dentin at an average 26 min exposure time [9]. A recent study showed that the increase in volume and or time of contact of 5.25% alkalized-NaOCl reduces the fracture strength of bovine teeth [10]. Zhang et al. suggested that the effect of NaOCl on mineralized dentin is both concentrations and time-dependent [11, 12]. In contrast, Hu et al. reported that time of exposures with is more important than the concentrations [13]. Structural changes caused by NaOCl as a root canal irrigant could compromise resin-dentin bond strength for tooth reconstruction [14–20]. Studies have shown that this effect of NaOCl is due to an oxygen-rich layer forming along the dentin surface followed by the breakdown of NaOCl into chlorine and oxygen. The remnants of the oxidative by-products interfere with the polymerization of adhesive cement and resin-based sealers [16–18]. Moghaddas et al. showed that the oxidizing effect of NaOCl could remain even two weeks after its application on dentin [21]. It suggests that applying an antioxidant solution such as STS or sodium ascorbate or long delay should be considered before the adhesive procedure to reverse this compromised bond strength [18, 22–24].

STS 5% is an antioxidant agent that recommends neutralizing the effect of NaOCl on dentin and improving the resin bonding properties [25]. It reacts with oxidants, which were produced by NaOCl to reduce unpaired electrons to form a stable product [26]. STS is used for medical conditions, such as calciphylaxis secondary to chronic renal failure, because of its chelating effect on calcium salts [27]. STS prevents calcification by chelating calcium and its acidosis-inducing properties [28]. STS also significantly reduces calcium-containing crystal formation in cultured murine chondrocytes. Administering STS decreases the volume and crystalline content of the new calcific deposits formed in the joint [29]. The articles about the STS bring forward to this section instead of discussion part.

The effects of this neutralizing agent on the physical properties of dentin are unknown. Thus, this in- vitro study evaluated the effect of STS on the micro-hardness of dentin treated with and without NaOCl at different time intervals. This study hypothesizes that STS will reduce the effect of NaOCl on dentin micro-hardness by neutralizing its remnants on the dentin surface after one week.

## Methods

### Preparation of tooth specimens

Fifty straight single-rooted teeth with relatively similar dimensions and morphology and closed apices were extracted for orthodontic or periodontal reasons collected with the patients´ informed consent. This study design was approved by the Ethics in Human Research Committee of Shiraz University of Medical Sciences (Ethics ID No. IR.SUMS.DENTAL.REC. 1398.138). Proximal view radiographs were taken to confirm the presence of a single patent canal. Teeth with root caries, cracks, curved canals, endodontic treatment, internal resorption, or calcification were excluded. Teeth were thoroughly cleaned of any soft tissue or calculus deposits and stored in isotonic saline solution at room temperature until the time of use. The crowns of all specimens were cut transversally at the coronal level of the roots with a double-faced diamond disc (Microdont, LDA, Brazil) at low speed with water coolant to ensure a uniform sample length of 14 ± 1 mm root length.

### Specimen preparation for the micro-hardness evaluation

Specimens were longitudinally sectioned in the buccolingual direction using a double-faced diamond disk at low speed, without passing through the canal space. A mallet and chisel were used to split the root. The root segments were horizontally embedded in auto polymerizing acrylic resin (Acrostone, Dent Product, Egypt), leaving their dentin surface exposed. The dentin surface of the mounted specimens was ground flat and smooth with a series of ascending grades of carbide abrasive papers (500, 800, 1000, and 1200 grit) (Bigo, Dent Product, Germany) under distilled water to remove any surface scratches and finally polished with a 0.1-Mm alumina suspension on a rotary felt disc (Microdont, LDA, Brazil) to obtain a smooth glassy mirror-like surface. The samples were divided randomly into one control and four experimental groups based on the immersion solution and incubation time:

Control group: Normal saline for 15 min, G1 and G2: 2.5% NaOCl (Chloraxid, Cerkamed, Poland) for 15 min, G3: 2.5% NaOCl for 15 min irrigated with normal saline followed by 5% STS (Merck, Darmstadt, Germany) for 10 min, G4: Normal saline for 15 min followed by 5% STS for 10 min. All groups except group 1 were incubated for 1 week in an incubator (37 °C with 100% humidity) before the micro-hardness test. The samples of group 1 were tested immediately after immersion in NaOCl.

### Dentin micro-hardness measurements

**Fig. 1:**
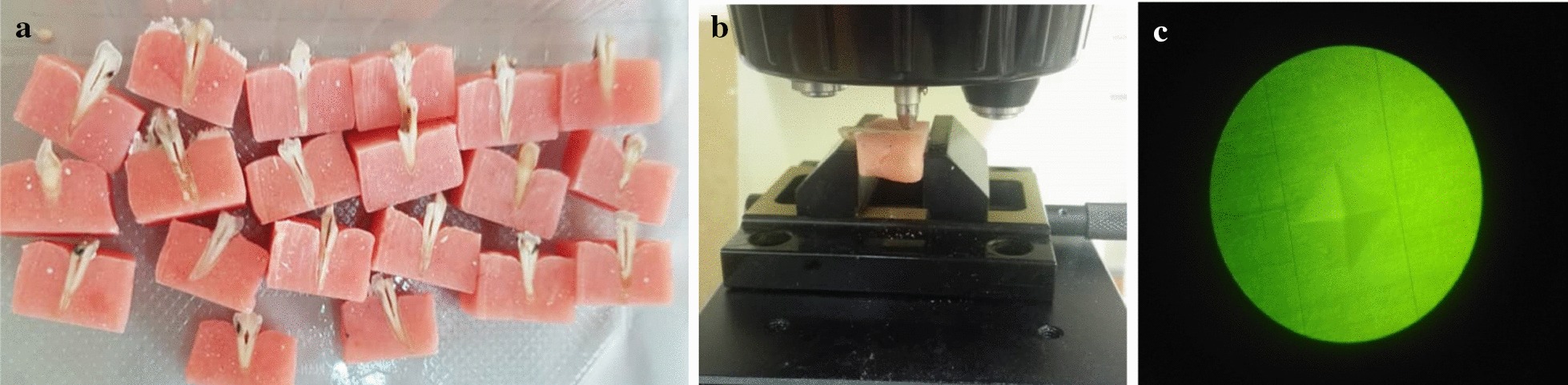
Some of prepared samples for test, **b** vickers testing machine **c** identation marks on dentin samples

The micro-hardness measurements were taken either on the buccal or lingual side of each root. The sectioned root was divided equally into three-thirds representing the coronal, middle and apical thirds, and each area was tested separately. An indentation was made in the dentin surface approximately 200 µm from the canal-dentin interface for standardization (Fig. 1). The Vickers hardness value was obtained by dividing the test force by the area of the sloping faces of the indentation. The resulting impression of the two diagonals was observed with an optical microscope and the average length of the two diagonals was measured with the built-in scaled micrometer and converted into the Vickers hardness number (VHN) with the following equation:

$${\text{VHN }}({\text{HV}}) = 1854\left( {{\text{F}}/{\text{D}}} \right)$$

The constant value of the equation was calculated from the specific geometry of the indenter, F is the applied load in grams and D is the diagonal of the indentation in μm [30].

### Specimen preparation for SEM evaluation

**Fig. 2:**
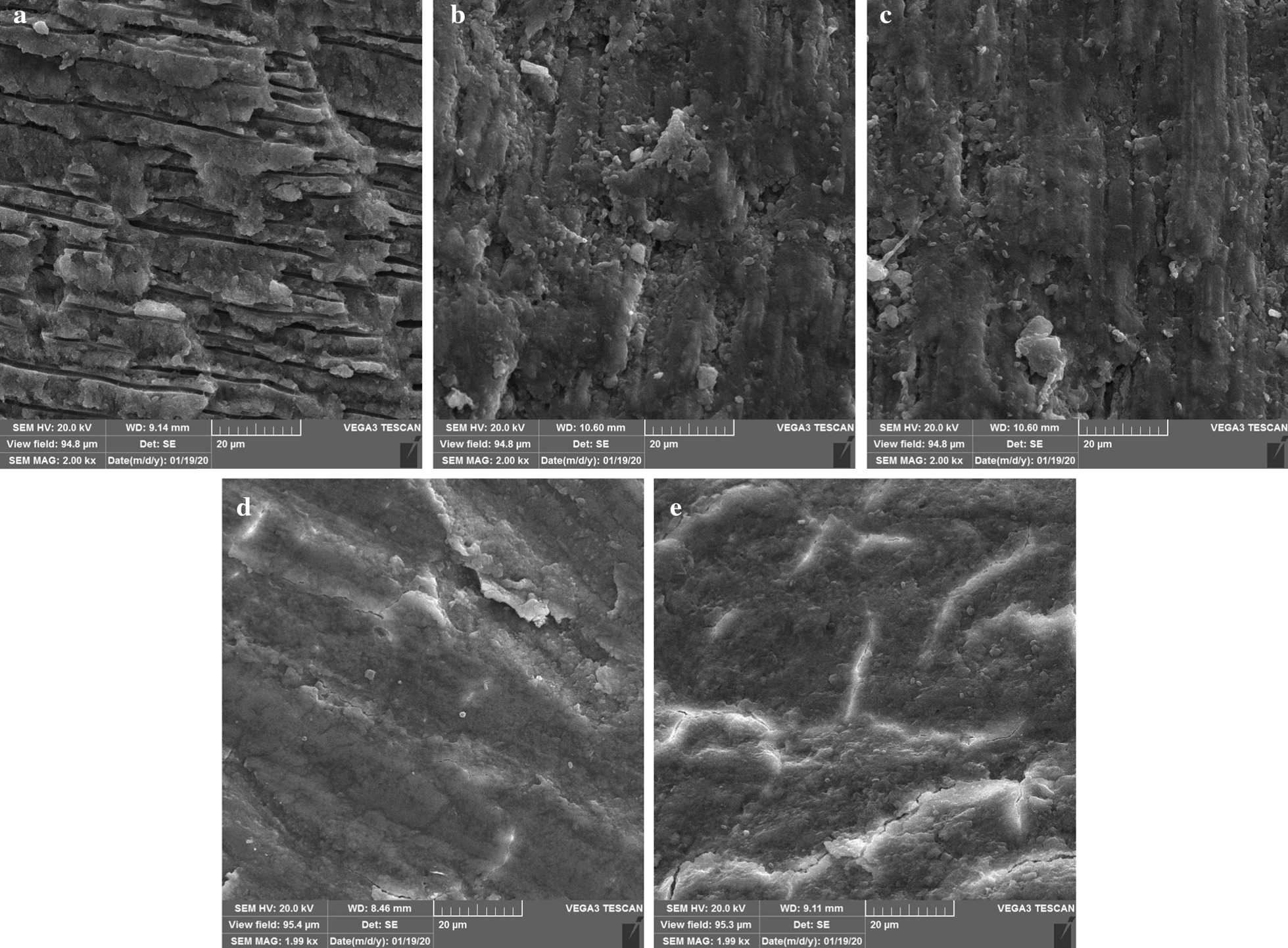
SEM microphotographs of the control sample at 2000× magnification. **a** Shows patent longitudinal dentinal tubule with patches of smear layers. Samples of NaOCl without incubation period and after one-week incubation **b**, **c** show dentin covered by smear layers with a few open dentinal tubes. The combination use of sodium thiosulfate and NaOCl or sodium thiosulfate alone **d**, **e** illustrated thicker and homogenous smear layers upon the dentin surface that covered all the dentinal tubules

One specimen of each group was dehydrated, mounted and gold-sputtered, for evaluation under a scanning electron microscope (Nova NanoSEM 450, FEI, Eindhoven, Netherlands) operated at 20KV. Photographs were taken from 3 points of each sample at 2000× magnifications (Fig. 2).

Data were analyzed using the Kruskal–Wallis test for pairwise comparisons. A *p* value < 0.05 was considered significant, and all analyses were carried out using SPSS software (SPSS version 16, SPSS INC., Chicago, IL, USA).

## Results

**Fig. 3:**
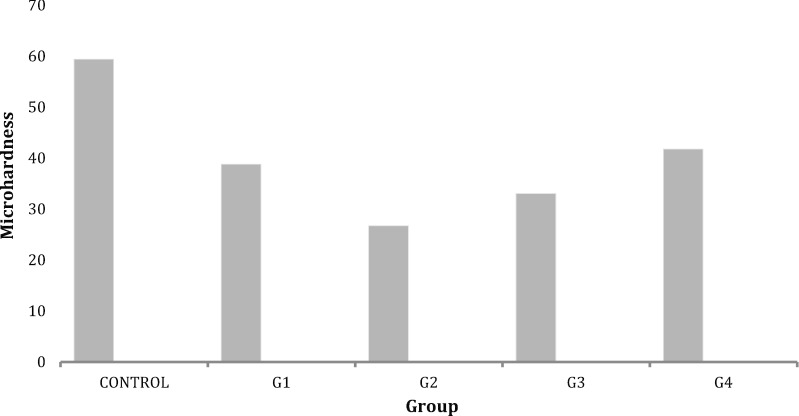
Microhardness of all experimental groups

The micro-hardness medians (means ± standard deviations) are shown in Table 1. The micro-hardness values of all of the groups decreased significantly compared with the control group (*p* < 0.05). As shown in Fig. 3, normal saline (control group) showed the highest micro-hardness while 2.5% NaOCl showed the lowest micro-hardness after 1 week (G2). A significant decrease in the micro-hardness value was observed between the samples tested immediately after NaOCl application (G1) and the samples incubated for 1 week (G2). Using NaOCl with STS together (G3) resulted in significantly lower micro-hardness than the control group and the STS alone group (G4) (*p* < 0.05).

**Table 1:** Median (Means ± Standard deviation) of microhardness in all experimental groups

Group	Median (mean ± SD)
0.9% NaCl for 15 min (control group) + 1-week incubation period	59.44 (59.40 ± 6.81)^a^
1. 2.5% NaOCL for 15 min + without incubation period	38.87 (39.44 ± 4.37)^bc^
2. 2.5% NaOCL for 15 min + 1-week incubation period	26.80 (28.02 ± 7.22)^d^
3. 2.5% NaOCL for 15 min irrigated with NaCl followed by 5% TS for 10 min + 1-week incubation period	33.12 (31.41 ± 4.80)^cd^
4. 0.9% NaCl for 15 min followed by 5% TS for 10 min + 1-week incubation period	41.83 (40.45 ± 6.70)^b^

The same superscript letters in the column are not statistically significant (*p* > 0.05)

SEM micrographs showed longitudinal dentinal tubes with the patches of some smear layers in the control group. In the other specimens, a unique pattern of smear layer, which is totally covered the dentin was obvious, although this layer seems thicker and more homogenous in the samples, which were in contact with STS.

The micro-hardness medians (means ± standard deviations) are shown in Table 1. The micro-hardness values of all of the groups decreased significantly compared with the control group (*p* < 0.05). As shown in Fig. 3, normal saline (control group) showed the highest micro-hardness while 2.5% NaOCl showed the lowest micro-hardness after 1 week (G2). A significant decrease in the micro-hardness value was observed between the samples tested immediately after NaOCl application (G1) and the samples incubated for 1 week (G2). Using NaOCl with STS together (G3) resulted in significantly lower micro-hardness than the control group and the STS alone (G4) (*p* < 0.05).

SEM micrographs showed longitudinal dentinal tubes with the patches of some smear layers in the control group. In the other specimens, a unique pattern of smear layer, which is totally covered the dentin was obvious, although this layer seems thicker and more homogenous in the samples, which were in contact with sodium thiosulfate.

## Discussion

The results of the present study show that both 2.5% NaOCl and STS decreased the micro-hardness of dentin compared with the control group. The remnants of NaOCl after 1 week significantly reduced the micro-hardness of dentin compared with the samples treated with NaOCl for 15 min.

Samples that were irrigated with NaOCl and then neutralized with STS, had significantly lower micro-hardness values than the samples irrigated with normal saline but were not different from the group immersed in NaOCl alone. Thus, STS not only reduced the effect of NaOCl on dentin micro-hardness but also may have had a synergistic effect on its weakening. Therefore, the hypothesis of this study was rejected.

Different studies have shown that using NaOCl as an irrigating solution significantly reduces the micro-hardness value [31–33]. Most studies used short exposure times of 5–15 min and revealed a reduction in the dentin micro-hardness value compared with irrigating with normal saline [32, 33]. Souza et al. reported that an increase in the volume and/or time of exposure to 5.25% NaOCl causes a significant reduction in root toughness. Even the increase in contact time without increasing the volume also negatively affects root toughness by about 37% [10]. Garcia et al. reported that 2.5% NaOCl, Chlor-XTRA, and 5.5% NaOCl gel all reduce the micro-hardness of dentin [34]. Slutzky-Goldberg et al. discovered that a 5 min exposure to 2.5% and 6% NaOCl does not reduce dentin micro-hardness but exposure for more than 10 min causes a significant reduction in micro-hardness. They also showed that 6% of NaOCl causes a more significant decrease in micro-hardness than the 2.5% concentration [35]. Thus, they suggested reducing the irrigation time to < 10 min and using a lower NaOCl concentration. Cochrane et al. exposed human dentine bars to 0.5% and 1% NaOCl gels and 1% and 4% NaOCl solutions for 7 days and then immediately subjected them to the Vickers micro-hardness test. Their results showed that the 0.5% NaOCl gel caused a significant decrease in the micro-hardness of the dentin bar [31]. The results of the present study agree with these studies, as the micro-hardness value decreased in the presence of NaOCl.

The interesting finding from this study is that the samples that were immersed for 15 min in NaOCl and irrigated with normal saline showed a significant reduction in micro-hardness after the one-week incubation. This finding indicates that the remnants of NaOCl and its oxidative products may remain active during this period leading to additional decreases in dentin micro-hardness. Concerning this subject, the use of an anti-oxidant agent like STS seems to be logical to reduce this effect. A recent study showed the recovery of resin bond strength to NaOCl treated dentin by using 5% STS for 10 min [25]. STS can react with NaOCl oxidants, to reduce them into a form a stable product and leads to better polymerization of resin [26]. In contrast to our hypothesis, STS alone or with NaOCl significantly decreased the micro-hardness value compared with the control group. It seems that neutralizing the oxidants of NaOCl with STS did not improve the micro-hardness of the dentin bars.

SEM micrographs also showed the same pattern of smear layers accumulation above the samples of NaOCl and STS (Fig. 2). This resemblance may explain the same effect of these materials on dentin micro-hardness. To the best of our knowledge, no study has evaluated the micro-hardness of dentin in the presence of STS or assessed the effect of STS on the micro-hardness of NaOCl treated dentin. STS is used in medicine for its chelating property in a situation like calciphylaxis secondary to chronic renal failure or reducing the crystalline content of the new calcific deposits formed in the joint [28, 29]. It seems, STS decreases the micro-hardness of dentin based on its chelating property and removing the calcium salts from the dentinal structure. Our results suggest that STS is not a suitable material for neutralizing NaOCl oxidant agents and may weaken dentin hardness. More studies are needed to confirm this result or to test other materials, such as sodium ascorbate, for this purpose.

## Conclusion

The reduction in dentine micro-hardness of all groups continued for one week even after irrigation the tested materials with normal saline. Neutralizing the remnants of NaOCl with STS did not prevent this adverse effect.

## Data Availability

The datasets used and/or analyzed during the current study are available from the corresponding author on reasonable request.

## Abbreviations

STS: Sodium thiosulfate
NaOCl: Sodium hypochlorite
SEM: Scanning electron microscope

## References

[1] Haikel Y, Gorce F, Allemann C, VOEGEL JC. In vitro efficiency of endodontic irrigation solutions on protein desorption. Int Endod J. 1994;27(1):16–20. 10.1111/j.1365-2591.1994.tb00223.x 7806406

[2] Pascon FM, Kantovitz KR, Sacramento PA, Nobre-dos-Santos M, Puppin-Rontani RM (2009). Effect of sodium hypochlorite on dentine mechanical properties: a review. J Dent..

[3] Sahebi S, Moazami F, Abbott P (2010). The effects of short-term calcium hydroxide application on the strength of dentine. Dent Traumatol.

[4] Sahebi S, Nabavizadeh M, Dolatkhah V, Jamshidi D (2012). Short term effect of calcium hydroxide, mineral trioxide aggregate and calcium-enriched mixture cement on the strength of bovine root dentin. Iran Endodontic journal.

[5] Moazami F, Sahebi S, Jamshidi D, Alavi A (2014). The long-term effect of calcium hydroxide, calcium-enriched mixture cement and mineral trioxide aggregate on dentin strength. Iran Endod J..

[6] Byström A, Sundqvist G. Bacteriologic evaluation of the effect of 0.5 percent sodium hypochlorite in endodontic therapy. Oral Surg Oral Med Oral Pathol. 1983;55(3):307–12. 10.1016/0030-4220(83)90333-x 6572884

[7] Grossman LI, Meiman BW (1941). Solution of pulp tissue by chemical agents. J Am Dent Assoc.

[8] Lee B-S, Hsieh T-T, Chi DC-H, Lan W-H, Lin C-P. The role of organic tissue on the punch shear strength of human dentin. J Dent. 2004;32(2):101–7. 10.1016/j.jdent.2003.09.001 14749081

[9] Souza EM, Calixto AM, e Lima CN, Pappen FG, De-Deus G. Similar influence of stabilized alkaline and neutral sodium hypochlorite solutions on the fracture resistance of root canal–treated bovine teeth. J. Endod. 2014;40(10):1600–3. 10.1016/j.joen.2014.02.028 25260730

[10] Souza EM, Quadros JdRP, Silva EJ, De-Deus G, Belladonna FG, Maia-Filho EM. Volume and/or time of NaOCl influences the fracture strength of endodontically treated bovine teeth. Braz Dent J. 2019;30(1):31–5. 10.1590/0103-6440201902076 30864644

[11] Zhang K, Kim YK, Cadenaro M, Bryan TE, Sidow SJ, Loushine RJ (2010). Effects of different exposure times and concentrations of sodium hypochlorite/ethylenediaminetetraacetic acid on the structural integrity of mineralized dentin. J Endod.

[12] Zhang K, Tay FR, Kim YK, Mitchell JK, Kim JR, Carrilho M (2010). The effect of initial irrigation with two different sodium hypochlorite concentrations on the erosion of instrumented radicular dentin. Dent Mater.

[13] Hu X, Peng Y, Sum C-p, Ling J. Effects of concentrations and exposure times of sodium hypochlorite on dentin deproteination: attenuated total reflection Fourier transform infrared spectroscopy study. J Endodo. 2010;36(12):2008–11. 10.1016/j.joen.2010.08.035 21092823

[14] Santos JN, de Oliveira Carrilho MR, De Goes MF, Zaia AA, de Almeida Gomes BPF, de Souza-Filho FJ (2006). Effect of chemical irrigants on the bond strength of a self-etching adhesive to pulp chamber dentin. J Endod.

[15] Farina AP, Cecchin D, Barbizam JV, Carlini-Júnior B (2011). Influence of endodontic irrigants on bond strength of a self-etching adhesive. Aust Endod J.

[16] Morris MD, Lee K-W, Agee KA, Bouillaguet S, Pashley DH (2001). Effects of sodium hypochlorite and RC-prep on bond strengths of resin cement to endodontic surfaces. J Endod.

[17] Erdemir A, Ari H, Güngüneş H, Belli S (2004). Effect of medications for root canal treatment on bonding to root canal dentin. J Endod.

[18] Lai S, Mak Y, Cheung G, Osorio R, Toledano M, Carvalho R (2001). Reversal of compromised bonding to oxidized etched dentin. J Dent Res.

[19] Francescantonio MD, Nurrohman H, Takagaki T, Nikaido T, Tagami J, Giannini M (2015). Sodium hypochlorite effects on dentin bond strength and acid-base resistant zone formation by adhesive systems. Braz J Oral Sci.

[20] Alkhudhairy FI, Yaman P, Dennison J, McDonald N, Herrero A, Bin-Shuwaish MS (2018). The effects of different irrigation solutions on the bond strength of cemented fiber posts. Clin Cosmet Investig Dent.

[21] Moghaddas MJ, Moosavi H, Ghavamnasiri M (2014). Microleakage evaluation of adhesive systems following pulp chamber irrigation with sodium hypochlorite. J Dent Res Dent Clin Dent Prospects.

[22] Vongphan N, Senawongse P, Somsiri W, Harnirattisai C (2005). Effects of sodium ascorbate on microtensile bond strength of total-etching adhesive system to NaOCl treated dentine. J Dent.

[23] Weston CH, Ito S, Wadgaonkar B, Pashley DH (2007). Effects of time and concentration of sodium ascorbate on reversal of NaOCl-induced reduction in bond strengths. J Endod.

[24] Prasansuttiporn T, Nakajima M, Kunawarote S, Foxton RM, Tagami J (2011). Effect of reducing agents on bond strength to NaOCl-treated dentin. Dent Mater.

[25] Corrêa ACP, Cecchin D, de Almeida JFA, de Almeida Gomes BPF, Zaia AA, Ferraz CCR (2016). Sodium thiosulfate for recovery of bond strength to dentin treated with sodium hypochlorite. J Endod.

[26] Aruoma OI, Cuppett SL. Antioxidant methodology: in vivo and in vitro concepts: the American Oil Chemists Society; 1997.

[27] Jost J, Bahans C, Courbebaisse M, Tran T-A, Linglart A, Benistan K (2016). Topical sodium thiosulfate: a treatment for calcifications in hyperphosphatemic familial tumoral calcinosis?. J Clin Endocrinol Metab..

[28] Pasch A, Schaffner T, Huynh-Do U, Frey BM, Frey FJ, Farese S (2008). Sodium thiosulfate prevents vascular calcifications in uremic rats. Kidney Int.

[29] Nasi S, Ea H-K, Lioté F, So A, Busso N (2016). Sodium thiosulfate prevents chondrocyte mineralization and reduces the severity of murine osteoarthritis. PLoS ONE.

[30] Massoud SF, Moussa SM, Hanafy SA, El Backly RM (2017). Evaluation of the microhardness of root canal dentin after different irrigation protocols (in vitro study). Alex. Dent J.

[31] Cochrane S, Burrow M, Parashos P (2019). Effect on the mechanical properties of human and bovine dentine of intracanal medicaments and irrigants. Aust Dent J.

[32] Marcelino APM, Bruniera JF, Rached-Junior FA, Silva SRCd, Messias DC. Impact of chemical agents for surface treatments on microhardness and flexural strength of root dentin. Braz Oral Res. 2014;28(1):1–6. 10.1590/1807-3107bor-2014.vol28.0052 25229790

[33] Aslantas EE, Buzoglu HD, Altundasar E, Serper A (2014). Effect of EDTA, sodium hypochlorite, and chlorhexidine gluconate with or without surface modifiers on dentin microhardness. J Endod..

[34] Garcia AJ, Kuga MC, Palma-Dibb RG, Só MV, Matsumoto MA, Faria G (2013). Effect of sodium hypochlorite under several formulations on root canal dentin microhardness. J Investig Clin Dent.

[35] Slutzky-Goldberg I, Maree M, Liberman R, Heling I (2004). Effect of sodium hypochlorite on dentin microhardness. J Endod.

